# A public-data audit of transcriptomic prognostic transportability in R-CHOP-treated diffuse large B-cell lymphoma

**DOI:** 10.1371/journal.pone.0360937

**Published:** 2026-10-23

**Authors:** Yongqing Li, Guowei Xiong, Wenhao Feng, Qi Chen, Yingying Qin, Haoyun Hong, Lin Lin

**Affiliations:** 1 Department of Hematology, Jieyang People’s Hospital, Jieyang, Guangdong, China; 2 Department of Laboratory Medicine, The Third Affiliated Hospital, Sun Yat-sen University, Guangzhou, Guangdong, China; 3 Pathology and Laboratory Center, Baiyun Hospital of The First Affiliated Hospital of Guangzhou University of Chinese Medicine, Guangzhou, Guangdong, China; 4 Department of Laboratory Medicine, Jieyang People’s Hospital, Jieyang, Guangdong, China; 5 Department of Gastroenterology, The First Affiliated Hospital of Shantou University Medical College, Shantou, Guangdong, China; 6 Jiexi County Second People’s Hospital, Jieyang, Guangdong, China; 7 Faculty of Medicine, School of Pharmacy, Medical Science Division, Macau University of Science and Technology, Macau SAR, China; 8 Department of Laboratory Medicine, Rongcheng District Central Hospital, Jieyang, Guangdong, China; Duke University Medical Center: Duke University Hospital, UNITED STATES OF AMERICA

## Abstract

Gene-expression biomarkers for diffuse large B-cell lymphoma (DLBCL) are often transported across retrospective cohorts that differ in treatment, assay platform, and clinical annotation. Using REMARK as the reporting framework, we audited prognostic associations with fixed scoring rules. Four Gene Expression Omnibus cohorts were harmonized for survival analysis. Treatment context was assessed after outcome-blind transcriptional state discovery. A directly mapped five-gene historical benchmark was evaluated with continuous Cox models in four R-CHOP cohorts. A 76-gene program selected in GSE31312 and GSE10846 served as a case study of two-cohort discovery, with GSE87371 as the only full-coverage independent evaluation and GSE32918 as a cross-platform sensitivity analysis. Coverage ablation, technical replicates, matched random programs, meta-analysis, and clinical-increment analyses assessed attenuation. The survival analyses included 470, 232, 87, and 140 patients. State composition was strongly associated with CHOP versus R-CHOP context in GSE10846 (Cramer’s V = 0.986). The historical score’s unadjusted random-effects hazard ratio (HR) was 1.35 (95% confidence interval 1.04–1.75); the prediction interval crossed the null (0.77–2.37). Common age/sex adjustment yielded HR 1.27 (1.05–1.54), with a prediction interval of 0.94–1.72. The 76-gene program was not independently validated in GSE87371 (unadjusted HR 1.23, 0.87–1.75; IPI/COO-adjusted HR 0.88, 0.56–1.40; age/sex-adjusted HR 1.09, 0.76–1.55). Its effect exceeded most matched random programs, but cross-cohort attenuation was pronounced and estimated clinical increment was negligible. With only 32 validation events, modest effects remain compatible with the data. Public DLBCL transcriptomic associations can be cohort-specific despite fixed scoring rules. Independent full-coverage evaluation and transparent treatment and platform audits should precede claims of general prognostic utility.

## Introduction

Diffuse large B-cell lymphoma (DLBCL) is clinically and molecularly heterogeneous. The addition of rituximab to CHOP chemotherapy improved outcomes, while gene-expression profiling identified cell-of-origin and microenvironmental programs associated with survival [1–4]. Low-dimensional expression models were subsequently proposed as practical prognostic tools [5,6]. Their clinical interpretation, however, depends on whether an association survives changes in treatment era, case mix, assay platform, and available clinical covariates.

Public DLBCL cohorts are repeatedly reused for biomarker discovery. This enables independent reanalysis, but it can also blur the distinction between derivation, contextual comparison, and external validation. A score may appear stable within one dataset while losing association after clinical adjustment or on a different platform. REMARK provides a reporting framework for prognostic-marker studies [7,8]. Here, the 76-gene program is a worked example of a conventional two-cohort discovery pipeline, not a proposed new clinical signature or an independently established biological program. It complements a literature-based benchmark derived from the Lossos and Malumbres six-gene models [5,6]; it does not replace evaluation of published models or represent all possible discovery pipelines.

We therefore performed an evidence-quality audit of prognostic associations. We first examined whether an outcome-blind transcriptomic partition was structurally coupled to CHOP versus R-CHOP context. We then assessed a historical low-dimensional benchmark and the derivation-selected 76-gene program across four public R-CHOP cohorts using fixed gene membership, directions, and scoring rules. Coverage ablation, technical-replicate resampling, and clinical positive controls were used to localize plausible sources of non-transportability.

## Materials and methods

### Study design and reporting

This retrospective secondary analysis used de-identified public data from the Gene Expression Omnibus (GEO) [9]. The analysis was organized as a transportability audit with explicit cohort roles. GSE31312 and GSE10846 were used during 76-gene program selection and were therefore not treated as independent validations. GSE87371 was the only independent cohort with complete 76-gene coverage. GSE32918 was restricted to a 62-of-76-gene sensitivity analysis. Reporting was guided by REMARK [7,8]. This study evaluates prognostic associations rather than developing or validating an individual-risk prediction model; TRIPOD compliance is not claimed. No new human participants were enrolled, and no new ethics approval or consent was required for analysis of public de-identified records. The source files used in the original analyses were accessed between 22 and 27 July 2026. The authors had access only to the de-identified records deposited in GEO and did not have access to information that could identify individual participants.

### Cohorts and endpoints

GSE31312 contained 498 expression arrays; 28 lacked a matched clinical record, leaving 470 overall-survival (OS)-eligible R-CHOP-treated patients (170 deaths) [10]. The clinical source contained 475 records, five of which could not be matched to an expression array. GSE10846 contained 420 arrays: six lacked treatment context, 181 were CHOP-treated, and 233 were R-CHOP-treated. One R-CHOP case lacked eligible OS follow-up, leaving 232 patients (59 deaths) [4]. GSE87371 contained 223 arrays; excluding 114 cases outside R-CHOP14/21 left 109, and excluding 22 without an ABC or GCB cell-of-origin (COO) classification left 87. These 87 had complete OS and International Prognostic Index (IPI) [11] data (32 deaths) [12]. GSE32918 contained 249 arrays representing 172 canonical patients plus 77 technical-replicate arrays. Excluding 32 patients not treated with curative intent left 140 R-CHOP-treated patients (62 deaths) for OS analysis [13]. No GSM identifier was shared between the four survival cohorts; this identifier-level check cannot exclude an unrecorded duplicate under different accessions. Cohort flow and covariate availability are detailed in Table 1 and Table A in S1 File.

**Table 1:** Cohort roles and primary overall-survival analyses.

Cohort	Analysis role	Platform	N (events)	Eligibility	Adjusted covariates
GSE31312	76-gene program derivation	GPL570	470 (170)	R-CHOP; OS eligible	Age, sex, stage, ECOG, COO
GSE10846	76-gene program derivation and treatment-context audit	GPL570	232 (59)	R-CHOP subset	Age, sex, stage, ECOG
GSE87371	Independent full 76-gene validation	GPL570	87 (32)	R-CHOP14/21; ABC/GCB	IPI, COO
GSE32918	62/76 cross-platform sensitivity and technical-replicate audit; not full validation	GPL8432 HumanRef-8 WG-DASL	140 (62)	R-CHOP treated	Age, sex, COO

Abbreviations: ABC, activated B-cell-like; COO, cell of origin; ECOG, Eastern Cooperative Oncology Group; GCB, germinal-center B-cell-like; IPI, International Prognostic Index; OS, overall survival.

### Expression processing and score construction

Verified GEO matrices and official platform annotations were used. Expression was mapped to Entrez Gene identifiers, and genes were standardized within each analytic cohort. The historical benchmark fixed LMO2, BCL6, and FN1 as favorable and CCND2 and BCL2 as adverse. CCL3 was excluded from the primary cross-platform benchmark because the GPL570 probe measured a CCL3/CCL3L family signal, whereas GPL8432 provided a direct CCL3 probe. The five-gene score was the mean signed within-cohort z score and was then standardized to one SD. This is a transparent, equal-weight cross-platform adaptation of the published gene set [5,6], not a reproduction of the original fitted six-gene prediction equations.

### Fixed 76-gene program and workflow provenance

Genome-wide screening used GSE31312 and the GSE10846 R-CHOP subset. Candidate genes required discovery false-discovery rate below 0.05, nominal P < 0.05 in the second cohort, and concordant direction. Exact adjusted Cox models were then fitted in both cohorts; 76 genes had nominal P < 0.05 and concordant adjusted direction in both. The program score was the mean of within-cohort standardized genes multiplied by the sign of the GSE31312 discovery coefficient. The supplied code derives membership and signs from these two cohorts and applies them unchanged in GSE87371 and GSE32918, without fitting validation-outcome weights or optimizing cutoffs. However, no independently verifiable, externally timestamped preregistration or pre-access code deposit was available. Code order and local file timestamps establish neither the date of first access to GSE87371 nor prospective locking. We therefore describe a retrospective audit of fixed derivation-based rules, rather than a prospectively registered validation. The label ‘locked’ in the figure schematics refers to computationally fixed membership and signs. Reviewer-requested common-covariate, direction-concordance, and power analyses were added during revision and are explicitly exploratory.

### Context, coverage, and technical audits

The outcome-blind state analysis used GSE10846 (DLBCL; 420 available arrays), GSE93261 (follicular lymphoma; 149 available arrays), and GSE21452 (mantle cell lymphoma; 64 arrays). Variance ranking and within-cohort standardization used these full discovery matrices. A balanced subset of 64 arrays from each cohort (192 total; seed 20260726) was used to fit PCA and k-means on the first six principal components of 2,000 variance-ranked genes; k = 4 was selected by silhouette score without using treatment or outcomes. Thus, the 64 GSE10846 fitting samples were nested within its 420-array series: 63 were among the 414 cases with known treatment, and 34 were among the 232 R-CHOP OS cases. States were assigned to all 420 GSE10846 arrays before treatment labels were joined. Because even the arrays outside the balanced fitting subset contributed to feature ranking and standardization, the state-by-treatment association is an in-sample descriptive audit, not an independent validation. GSE31312 and GSE32018 were used for external state projection/recovery only in this analysis; GSE31312 separately served as a survival-program derivation cohort. Table R in S1 File summarizes these roles, and S1 Data provides the complete GSM crosswalk. In GSE87371, the actual 62 genes measurable in GSE32918 were compared with the full 76-gene score and with 1,000 uniform and 1,000 mean/variance-matched random 62-gene subsets (seed 20260727). In GSE32918, canonical arrays were compared with the mean of available replicates, and 1,000 analyses randomly selected one array per patient.

### Statistical analysis

Primary score analyses used continuous Cox proportional-hazards models per 1-SD higher score with Breslow handling of ties and two-sided Wald tests. The original adjusted models used age, sex, stage, ECOG performance status, and COO in GSE31312; age, sex, stage, and ECOG in GSE10846; IPI and COO in GSE87371; and age, sex, and COO in GSE32918. These cohort-specific adjusted estimates were not pooled. Unadjusted coefficients were combined by DerSimonian-Laird random effects with Hartung-Knapp-Sidik-Jonkman inference, a 95% prediction interval, leave-one-cohort-out analyses, and influence diagnostics. A revision sensitivity analysis fitted the same age (continuous, per decade) and sex covariates in all four cohorts without changing eligibility or score scaling, then applied the same pooling method. All 929 OS-eligible cases had these covariates. The four-cohort 76-gene summary includes derivation cohorts and the mapped-subset cohort and is a heterogeneity diagnostic, not independent validation. A one-stage Cox model stratified by cohort tested score-by-cohort interaction. Median-split Kaplan-Meier curves were visual only. Agreement analyses used Spearman correlation, consistency ICC(3,1), quartile agreement, and top-quartile overlap. IPI and ABC-versus-GCB effects served as analysis-pipeline positive controls.

Except for the false-discovery-rate filter used in the initial genome-wide selection, no multiplicity correction was applied across the subsequent audit analyses. These analyses address related diagnostic questions rather than a single confirmatory hypothesis family; their P values are nominal and exploratory, and interpretation emphasizes effect sizes, confidence and prediction intervals, and cross-cohort consistency. Random-program percentiles are descriptive reference measures rather than multiplicity-adjusted significance claims.

During revision, gene-direction concordance was compared with a 0.5 null using two-sided exact binomial tests. Because genes are correlated, these tests provide a descriptive benchmark under an independence assumption rather than a correlation-adjusted confirmatory test. Validation-cohort power was contextualized using an event-based normal approximation for a continuous score of unit variance [14], with 32 deaths, two-sided alpha = 0.05, and a noncentrality parameter of log(HR) multiplied by the square root of 32. We evaluated the derivation effects and smaller hypothetical effects and estimated the HR detectable with 80% power; these calculations do not use the validation point estimate as the assumed true effect.

### Matched random programs and clinical increment

One thousand 76-gene random programs were assembled without survival outcomes. For each fixed gene position, a replacement gene was sampled from the 25 nearest genes by GSE87371 mean expression and log variance while preserving GPL8432 mapping status, the 62-of-76 mapping count, and the fixed 61 favorable/15 adverse sign pattern. The fixed and random programs were scored identically in all four cohorts. We compared the fixed program with the null distributions of the GSE87371 HR, I2, and derivation-to-GSE87371 coefficient attenuation. Clinical-only and clinical-plus-score Cox models were compared within each cohort by likelihood-ratio test and change in Harrell’s C-index; 300 bootstrap samples estimated optimism in the C-index increment. These analyses were association-level diagnostics and not clinical prediction-model validation. Core analyses used Python 3.12.13; the extension used R 4.3.2 with survival 3.8.3.

## Results

### Cohort provenance and treatment context

The final OS cohorts contained 470, 232, 87, and 140 patients, with no cross-cohort GSM overlap (Fig 1A-C). GSE32918 required explicit separation of 172 canonical patients from 77 noncanonical replicate arrays. The balanced state-fitting set included 64 GSE10846 samples, of which 34 also entered the R-CHOP OS analysis (Table R in S1 File). In the full GSE10846 state assignment, 178 of 181 CHOP-treated cases were in S2, whereas 180 of 233 R-CHOP cases were in S3 and 53 were in S4. State composition was strongly associated with known treatment context (n = 414; chi-square = 402.5; P = 4.0x10^-88; Cramer’s V = 0.986; Fig 1D). Because treatment was not randomized, the state model used this cohort’s expression data, and treatment labels were introduced after clustering, this result describes structural cohort coupling, not a treatment-effect estimate or independent replication.

**Fig 1:**
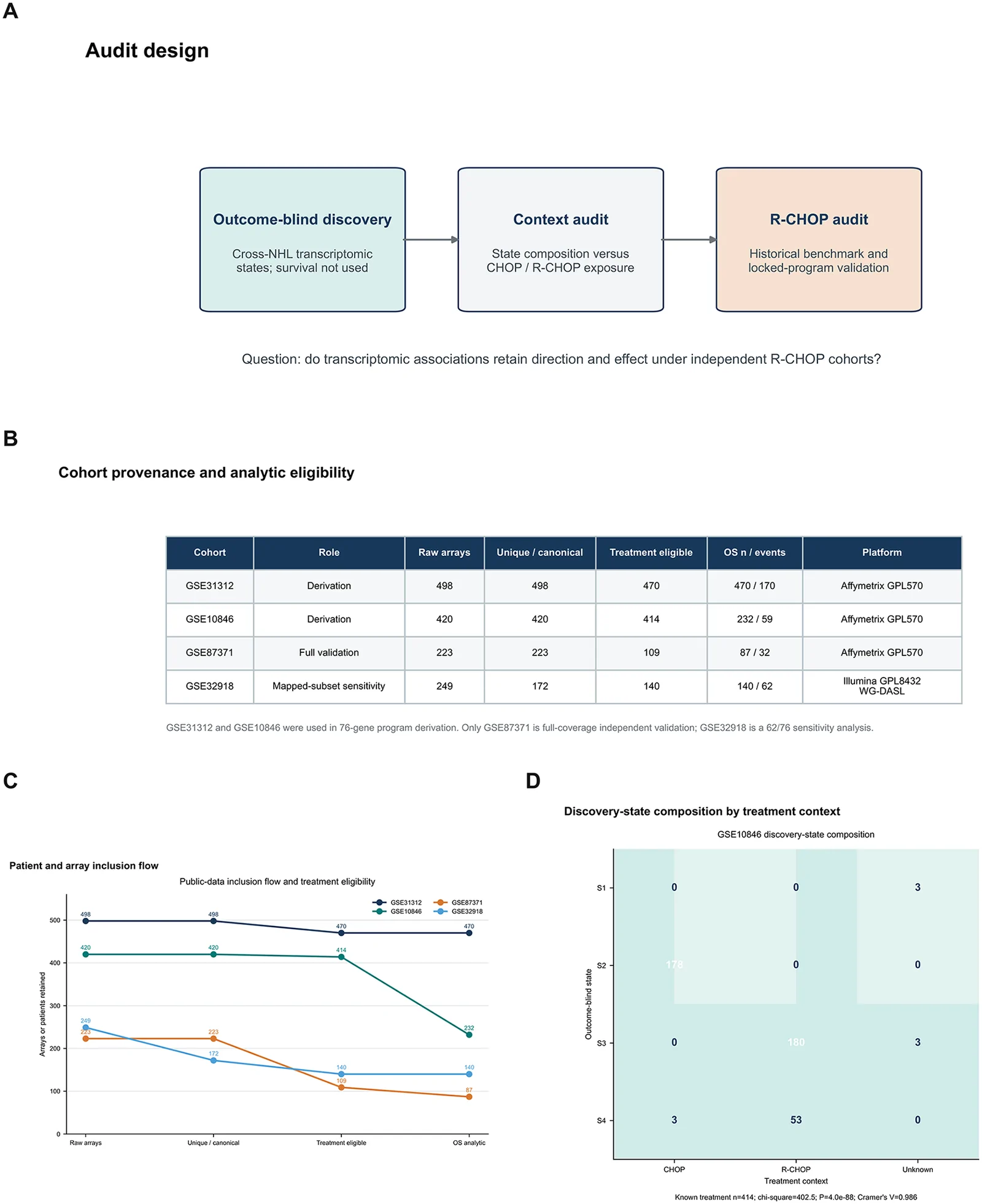
Cohort provenance and treatment context. (A) Audit design separating outcome-blind state discovery, context assessment, and R-CHOP-focused survival analysis. (B) Provenance, role, platform, and analytic eligibility of the four DLBCL survival cohorts. (C) Sequential patient and array flow: GSE31312, 498 arrays minus 28 without matched clinical records gives 470 OS cases (475 clinical records included five without a matched array); GSE10846, 420 arrays minus six with unknown treatment gives 414 for the context audit, of which 233 were R-CHOP-treated and 232 had eligible OS after one further exclusion; GSE87371, 223 arrays minus 114 outside R-CHOP14/21 gives 109, then minus 22 without ABC/GCB classification gives 87; GSE32918, 249 arrays minus 77 noncanonical technical replicates gives 172 canonical patients, then minus 32 not treated with curative intent gives 140. (D) GSE10846 state-by-treatment counts after treatment labels were introduced post hoc. The association (n = 414; chi-square = 402.5; P = 4.0x10^-88; Cramer’s V = 0.986) is an in-sample cohort-context audit, not a causal treatment-effect estimate.

### Historical five-gene benchmark shows heterogeneous transportability

The direct five-gene score was associated with worse OS in GSE31312 (unadjusted HR 1.39, 95% CI 1.21–1.61; adjusted HR 1.22, 95% CI 1.03–1.44), GSE10846 R-CHOP (unadjusted HR 1.45, 95% CI 1.12–1.88; adjusted HR 1.25, 95% CI 0.95–1.65), and GSE87371 (unadjusted HR 1.63, 95% CI 1.14–2.33; adjusted HR 1.41, 95% CI 0.78–2.56). The association was null in GSE32918 (unadjusted HR 1.05, 95% CI 0.82–1.36; adjusted HR 0.96, 95% CI 0.72–1.27; Fig 2 and Table 2). The Hartung-Knapp random-effects estimate was HR 1.35 (95% CI 1.04–1.75; P = 0.037; I2 = 41.3%), while the 95% prediction interval was 0.77–2.37. With common age/sex adjustment, the pooled HR was 1.27 (95% CI 1.05–1.54; P = 0.027; I2 = 8.8%), and the prediction interval remained compatible with no association in a new cohort (0.94–1.72; Tables S and T in S1 File). Thus, the average association was not solely attributable to different adjustment sets, but its transportability remained uncertain. Excluding the ambiguous CCL3-family measurement did not remove the GSE32918 discrepancy.

**Table 2:** Continuous-score overall-survival associations.

Score	Cohort	Role	Coverage	Model	N (events)	HR/SD	95% CI	P
Historical five-gene	GSE31312	Benchmark	5/5	Unadjusted	470 (170)	1.39	1.21-1.61	5.87e-06
Historical five-gene	GSE31312	Benchmark	5/5	Adjusted	470 (170)	1.22	1.03-1.44	0.0194
Historical five-gene	GSE10846 R-CHOP	Benchmark	5/5	Unadjusted	232 (59)	1.45	1.12-1.88	0.00528
Historical five-gene	GSE10846 R-CHOP	Benchmark	5/5	Adjusted	206 (52)	1.25	0.95-1.65	0.114
Historical five-gene	GSE87371	Benchmark	5/5	Unadjusted	87 (32)	1.63	1.14-2.33	0.00746
Historical five-gene	GSE87371	Benchmark	5/5	Adjusted	87 (32)	1.41	0.78-2.56	0.254
Historical five-gene	GSE32918	Benchmark	5/5	Unadjusted	140 (62)	1.05	0.82-1.36	0.687
Historical five-gene	GSE32918	Benchmark	5/5	Adjusted	140 (62)	0.96	0.72-1.27	0.758
Fixed 76-gene	GSE87371	Independent validation	76/76	Unadjusted	87 (32)	1.23	0.87-1.75	0.243
Fixed 76-gene	GSE87371	Independent validation	76/76	Adjusted	87 (32)	0.88	0.56-1.40	0.599
Mapped 62/76	GSE32918	Sensitivity only	62/76	Unadjusted	140 (62)	1.13	0.89-1.43	0.307
Mapped 62/76	GSE32918	Sensitivity only	62/76	Adjusted	140 (62)	1.08	0.83-1.41	0.55

**Fig 2:**
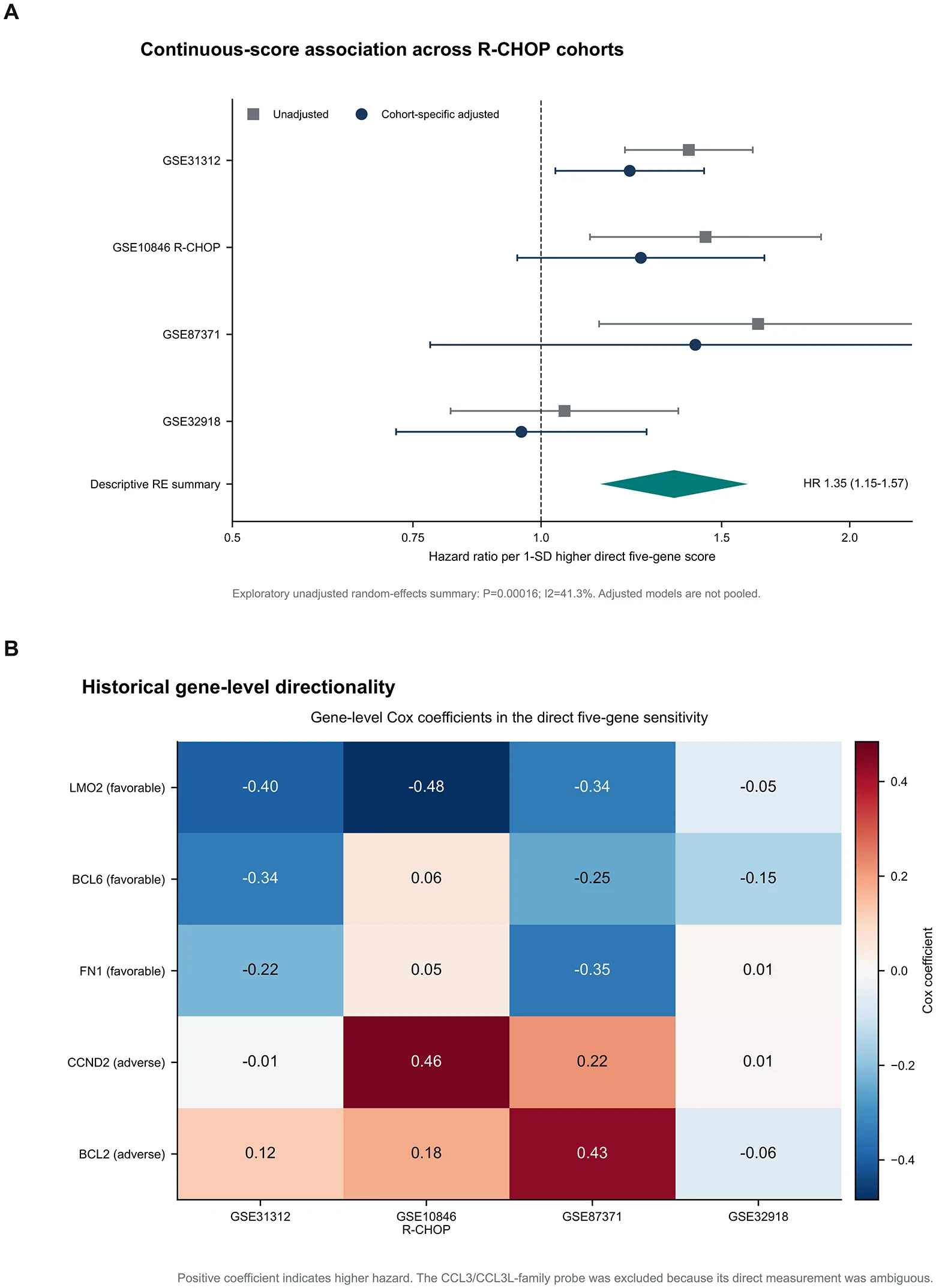
Transportability of the historical direct five-gene benchmark. (A) Continuous Cox associations per 1-SD higher score. Squares show cohort estimates and the diamond shows the exploratory unadjusted DerSimonian-Laird random-effects summary. The original cohort-specific adjusted estimates were not pooled because their covariate sets differed; a separate common age/sex-adjusted sensitivity analysis is reported in Tables S and T in S1 File. (B) Gene-level Cox coefficients across cohorts. The score includes LMO2, BCL6, FN1, CCND2, and BCL2; CCL3 was excluded because its GPL570 assay was a CCL3/CCL3L-family probe.

Adjusted covariates were cohort specific as listed in Table 1. GSE32918 evaluates only the mapped 62-of-76-gene subset.

### The fixed 76-gene program is not independently validated

The 76-gene program was evaluated after two-cohort selection, with GSE87371 as the only full-coverage independent cohort (Fig 3A-D). In GSE87371, the program was not associated with OS in the unadjusted model (HR 1.23, 95% CI 0.87–1.75; P = 0.243) or after IPI and COO adjustment (HR 0.88, 95% CI 0.56–1.40; P = 0.599). Common age/sex adjustment also yielded an imprecise estimate near the null (HR 1.09, 95% CI 0.76–1.55; P = 0.638). GSE32918 mapped 62 of 76 genes (81.6%) and was analyzed only as a sensitivity cohort; its unadjusted HR was 1.13 (95% CI 0.89–1.43; P = 0.307), original adjusted HR was 1.08 (95% CI 0.83–1.41; P = 0.550), and age/sex-adjusted HR was 1.08 (95% CI 0.86–1.37; P = 0.510). Heterogeneity remained high in the diagnostic four-cohort age/sex-adjusted summary (I2 = 92.7%; Tables S and T in S1 File). Gene-level directions agreed for 44/76 genes in GSE87371 (57.9%; two-sided exact binomial P = 0.207) and 38/62 in GSE32918 (61.3%; P = 0.098), neither nominally different from 0.5 (Fig 3C, S3 Fig, and Table U in S1 File). These findings do not support advancing this program as a general R-CHOP prognostic signature; they do not establish equivalence to no effect.

**Fig 3:**
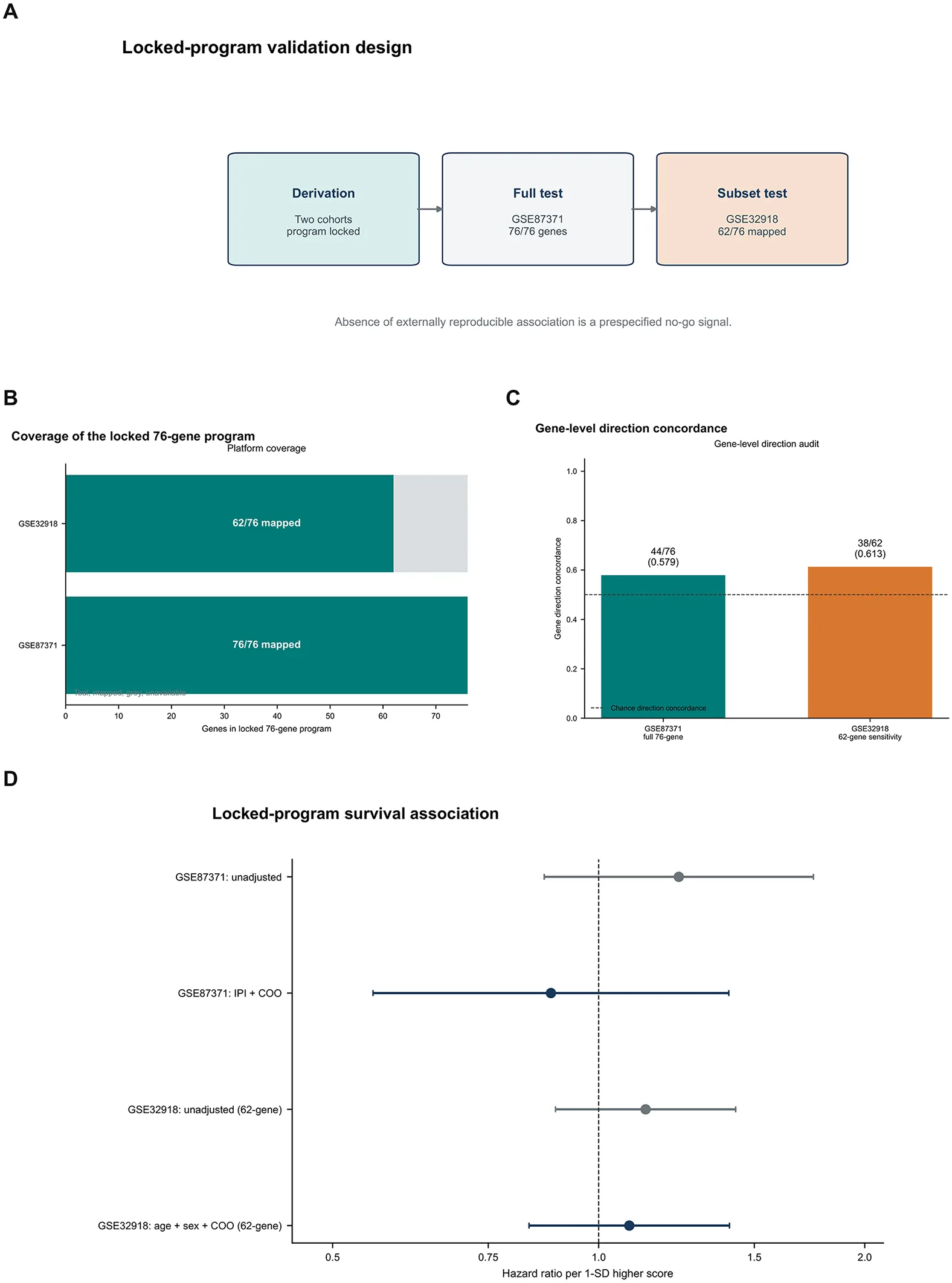
Independent evaluation of the fixed 76-gene program. (A) Evaluation design: GSE31312 and GSE10846 were used during program selection; GSE87371 was the only full-coverage independent cohort; GSE32918 was a mapped-subset sensitivity analysis. (B) Platform coverage. (C) Gene-level direction concordance: 44/76 (57.9%) in GSE87371 and 38/62 (61.3%) in GSE32918; two-sided exact binomial tests against 0.5 yielded nominal P = 0.207 and P = 0.098, respectively. These descriptive tests assume independent gene signs and do not account for gene correlation. (D) Continuous Cox associations per 1-SD higher score. The full-coverage and mapped-subset results did not provide independent support for general prognostic use; modest effects remain possible.

### Coverage ablation does not explain the validation result

Within GSE87371, the actual GSE32918-mapped 62-gene score closely tracked the full 76-gene score (Spearman rho = 0.990; ICC[3,1]=0.992; quartile agreement = 90.8%; top-quartile overlap = 83.3%; Fig 4A). The 62-gene unadjusted HR was 1.19 (95% CI 0.84–1.68), close to the full-program HR of 1.23 (Fig 4B). Its change in log HR was at the 20th percentile of uniformly random 14-gene deletions and the 17.5th percentile of mean/variance-matched deletions (Fig 4C and 4D). Thus, incomplete coverage perturbed the score modestly but was not an exceptional deletion pattern.

**Fig 4:**
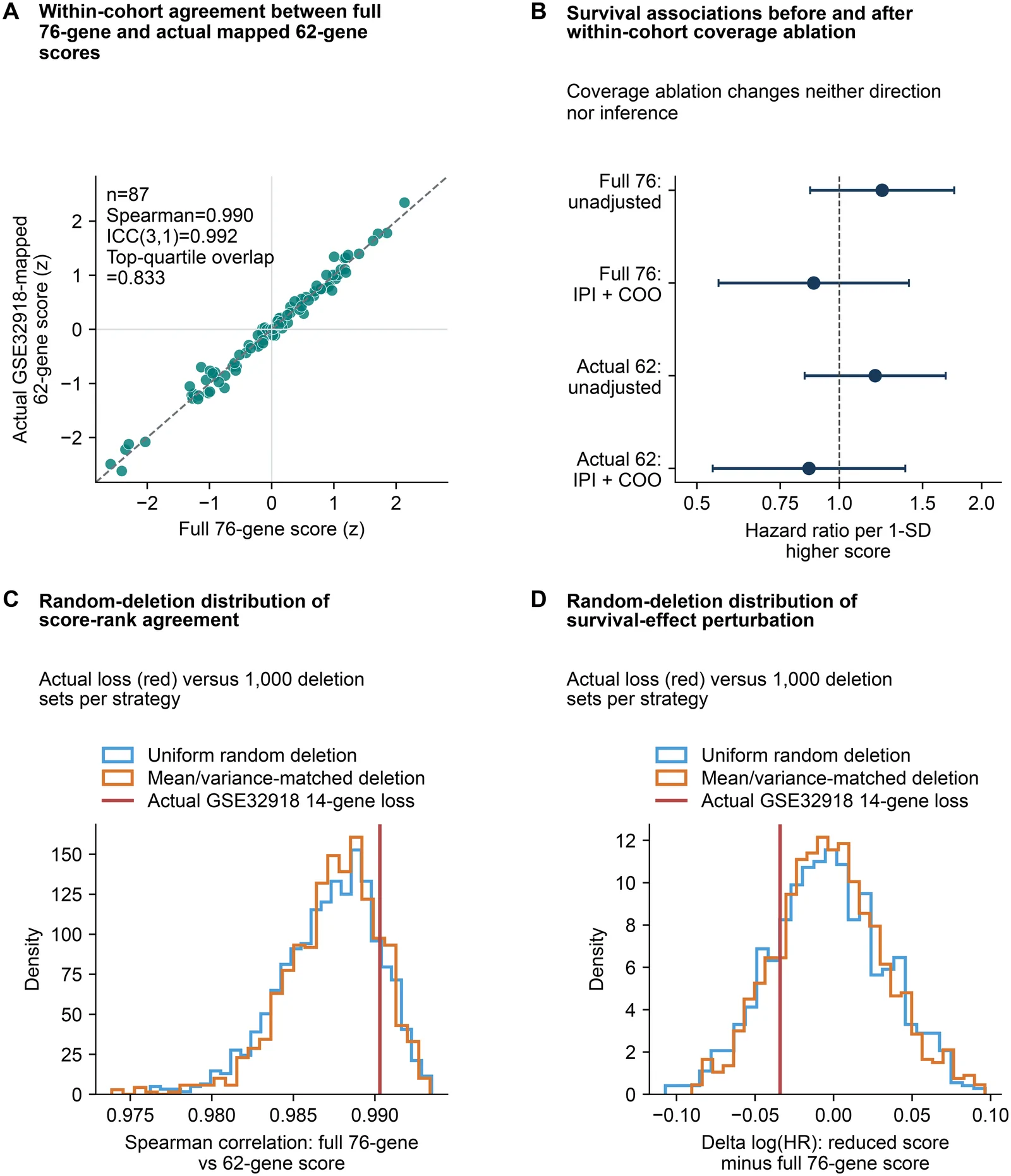
Within-cohort platform coverage ablation. (A) Agreement between the full 76-gene score and the actual 62-gene subset measurable on GSE32918, evaluated within GSE87371. (B) Unadjusted and IPI/COO-adjusted Cox estimates before and after ablation. (C,D) The actual mapped subset (red line) compared with 1,000 uniform random and 1,000 mean/variance-matched 62-of-76-gene subsets. Panel C shows score-rank agreement; panel D shows change in log HR relative to the full score.

### Technical variation is present but does not explain the weak association

Thirty-nine GSE32918 patients had at least one technical-replicate array. Agreement between canonical scores and mean replicate scores was moderate (ICC[3,1]=0.611; Pearson r = 0.664), and the median gene-level ICC was 0.535 (Fig 5A, 5B and 5D). Across 1,000 random one-array-per-patient analyses, the median unadjusted HR was 1.112 and the 2.5th-97.5th percentile range was 1.062–1.169 (Fig 5C), which included the canonical-array estimate near 1.13. Expected-direction positive controls remained detectable: IPI HRs per point were 1.69 in GSE31312 and 2.12 in GSE87371; ABC-versus-GCB HRs ranged from 1.76 to 3.60 across three cohorts (S4 Fig).

**Fig 5:**
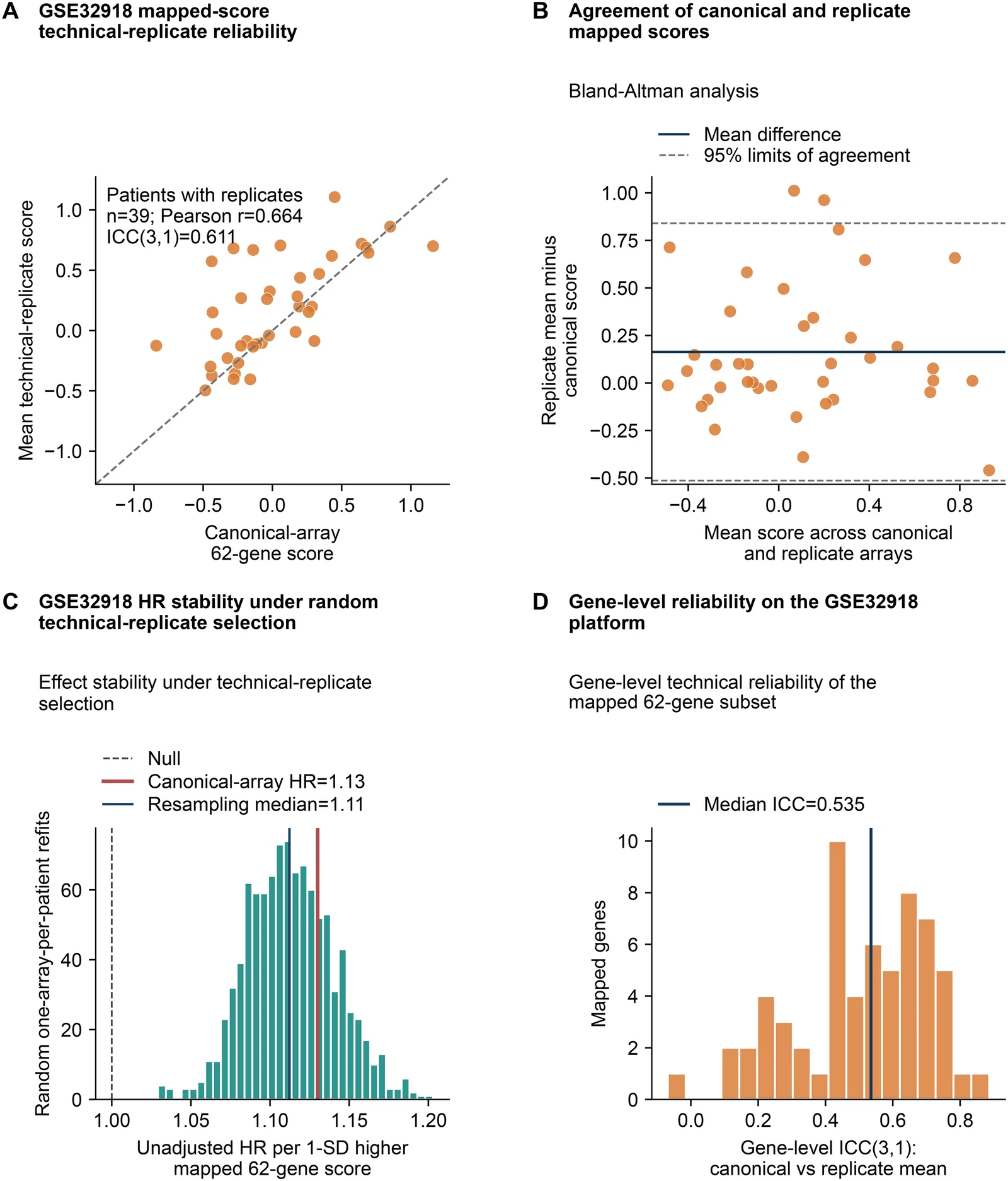
GSE32918 technical-replicate audit. (A) Canonical-array versus mean-replicate score agreement among 39 patients with replicates. (B) Bland-Altman assessment of paired scores. (C) Distribution of unadjusted OS HRs across 1,000 random one-array-per-patient selections; the canonical-array estimate is indicated. (D) Gene-level ICC(3,1) values for the 62 mapped genes.

### Expanded null and incremental analyses confirm attenuation

The one-stage stratified Cox model estimated a common historical five-gene HR of 1.35 (95% CI 1.22–1.51) without statistically detectable score-by-cohort interaction (likelihood-ratio P = 0.162; Fig 6). GSE32918 contributed 72.0% of Cochran’s Q; omitting it reduced I2 to 0% and yielded an HKSJ pooled HR of 1.43 (95% CI 1.23–1.65), whereas the other three leave-one-out intervals crossed 1. The fixed 76-gene program’s GSE87371 HR was at the 96.8th percentile of 1,000 outcome-blind matched random programs, but its I2 was 92.4% (100th percentile) and its derivation-to-GSE87371 log-HR attenuation was 0.678 (99.5th percentile; S5 Fig). Clinical-plus-score models showed no consistent improvement (S6 Fig). Only the GSE31312 historical-score model improved fit at nominal P < 0.05; its bootstrap-corrected C-index increment was 0.008. For the fixed program, corrected increments were −0.004 in GSE87371 and −0.007 in GSE32918. These changes of less than 0.01 are near zero and indicate negligible estimated incremental discrimination, not evidence that adding the score causes clinical harm.

**Fig 6:**
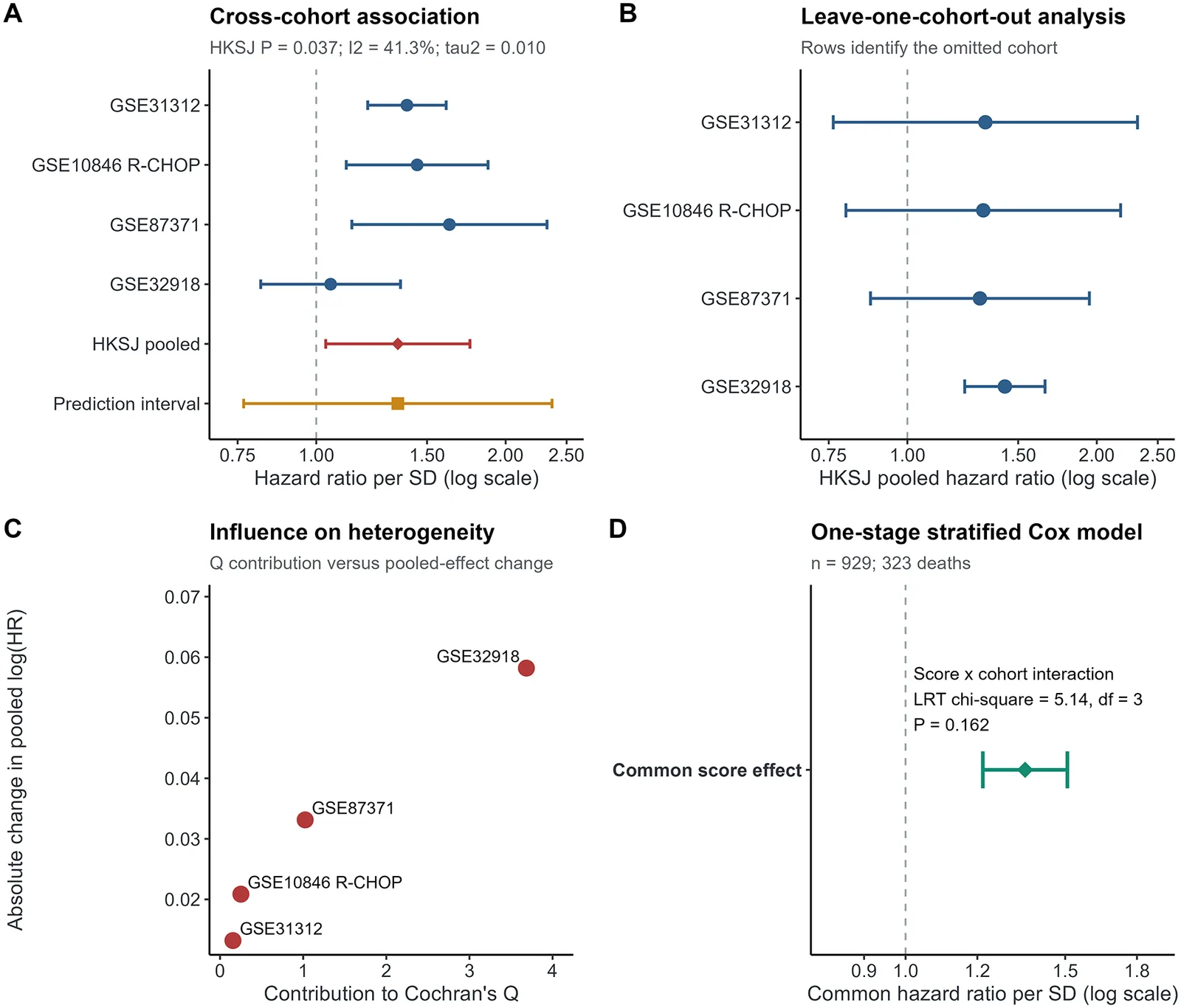
Expanded meta-analysis and heterogeneity audit of the historical five-gene score. (A) Cohort-specific unadjusted Cox estimates, Hartung-Knapp-Sidik-Jonkman random-effects summary, and 95% prediction interval. (B) Leave-one-cohort-out HKSJ estimates; rows identify the omitted cohort. (C) Cohort contributions to Cochran’s Q versus the absolute change in pooled log HR when omitted. (D) Common score effect from a one-stage Cox model stratified by cohort and likelihood-ratio test of score-by-cohort interaction.

## Discussion

This public-data audit separates evidence of an average association from evidence of prognostic transportability. The literature-based five-gene benchmark retained a positive unadjusted association in three cohorts, and its pooled association persisted with common age/sex adjustment. However, both unadjusted and common-adjustment prediction intervals crossed the null, the GSE32918 result was discordant, and clinical incremental value was limited. The derivation-selected 76-gene case study did not obtain support in the only independent full-coverage cohort. Its weak external association persisted with the same age/sex adjustment used in the derivation cohorts. Heterogeneous adjustment sets therefore do not fully explain the observed attenuation. The conclusion is incomplete transportability in these data, not a new clinical signature or proof that all expression-based prognostic programs lack value.

The analyses localize several constraints. First, treatment context can be embedded in transcriptomic structure: outcome-blind states in GSE10846 almost separated CHOP from R-CHOP. This does not identify a treatment-response biomarker, but it shows why mixed historical treatment cohorts should not be interpreted as exchangeable. Second, the within-GSE87371 ablation preserved score rank and effect size after removal of the 14 genes unavailable in GSE32918. Platform coverage matters, but the specific missing-gene set was not sufficient to explain the weak cross-platform association. Third, technical replicate variation was measurable, yet random array selection produced a narrow distribution centered near the canonical estimate. The matched-program null added an important qualification: the fixed program’s independent-cohort effect was stronger than most expression- and coverage-matched random programs, but its cross-cohort heterogeneity and derivation-to-external attenuation were more extreme. A non-random signal can therefore remain insufficiently transportable.

These findings have practical implications for public-database biomarker studies. Derivation cohorts should be named as such even when they are used sequentially. A mapped-subset analysis should not be relabeled as full external validation. Continuous score effects and clinical adjustment should remain primary; optimized cutoffs and Kaplan-Meier separation can otherwise overstate evidence. Clinical positive controls help check endpoint and covariate behavior, but their detection does not establish adequate power for every molecular effect. Adding the fixed score did not provide consistent likelihood-ratio or optimism-corrected C-index improvement. The corrected increments of −0.004 and −0.007 are practically near zero and are consistent with negligible incremental discrimination at the precision available, rather than deterioration in patient outcomes.

Limitations include retrospective public cohorts, heterogeneous eligibility and follow-up, incomplete clinical covariates, and one independent full-coverage cohort. Common age/sex adjustment improves comparability but is less comprehensive than adjustment for IPI, stage, ECOG, and COO and cannot eliminate residual confounding. GSE87371 had only 87 patients and 32 deaths. Under a two-sided 0.05 event-based approximation for a standardized continuous score [14], power to detect the derivation HRs of 2.28 and 2.88 was 99.7% and greater than 99.9%, respectively; power for their inverse-variance summary HR of 2.43 was 99.9%. These selection-enriched derivation effects are likely optimistic. For smaller true HRs of 1.30 and 1.50, approximate power was only 31.7% and 63.1%; 80% power required HR about 1.64 (Table V in S1 File). These scenarios assume proportional hazards, unit score variance, and non-informative censoring; they are not an observed-power test or proof of absence of association. The GSE87371 unadjusted interval (0.87–1.75) remains compatible with modest adverse effects. The state analysis was exploratory, included expression data from the same cohort used for the treatment-context audit, and supports neither independent replication nor causal treatment inference. No externally verifiable pre-access registration or code timestamp was available, so computationally fixed rules should not be interpreted as documented prospective locking. Harrell’s C-index was used only as an internal incremental diagnostic with limited bootstrap correction; calibration, time-dependent discrimination, decision-curve utility, and prospective validation were not assessed.

In conclusion, transcriptomic prognostic associations in R-CHOP-treated DLBCL can attenuate across cohorts despite fixed derivation-based scoring rules and targeted technical audits. The present results do not establish general prognostic utility and do not exclude smaller effects in larger independent cohorts. Full-coverage independent evaluation, transparent treatment eligibility, and explicit platform audits should precede clinical prognostic claims.

## Supporting information

S1 File Supplementary methods and tables. The file contains supplementary methods and Tables A-V.
(DOCX)

S1 Fig Within-discovery stability and external state recovery. (A) Median bootstrap stability of the outcome-blind cross-NHL and R-CHOP-restricted discovery analyses. (B) Bootstrap distributions of mean state Jaccard. (C) Mean matched centroid correlations after de novo clustering of external cohorts. Stability and external recovery are different metrics and are not directly interchangeable.
(TIF)

S2 Fig Median-split survival visualizations. Kaplan-Meier curves for the historical direct five-gene score in GSE31312, GSE10846 R-CHOP, GSE87371, and GSE32918. Median splits are shown for visualization only; continuous Cox models are the primary inference.
(TIF)

S3 Fig Gene-level direction concordance of the fixed program. Discovery coefficients are compared with GSE87371 full-program coefficients and GSE32918 mapped-subset coefficients. Teal points indicate concordant coefficient signs and red points indicate discordant signs. Concordance was 44/76 and 38/62, with descriptive two-sided exact binomial P = 0.207 and P = 0.098 against a 0.5 null proportion, respectively; gene dependence is not accounted for by these tests.
(TIF)

S4 Fig Clinical positive controls. Univariable Cox estimates for IPI per point and ABC versus GCB. Controls were used to verify expected endpoint and covariate behavior and were not pooled or used for gene-program selection.
(TIF)

S5 Fig Outcome-blind matched random-program null. The fixed 76-gene program is compared with 1,000 random 76-gene programs matched by GSE87371 mean expression, log variance, GPL8432 mapping status, and fixed sign position. (A) GSE87371 HR. (B) Cross-cohort I2. (C) Difference between the derivation-cohort and GSE87371 log HRs. (D) Derivation-cohort versus GSE87371 log HR. The fixed program’s percentiles were 96.8, 100.0, and 99.5 for panels A-C, respectively.
(TIF)

S6 Fig Clinical-only versus clinical-plus-score analyses. (A) Score associations after cohort-specific clinical adjustment. (B) Apparent and 300-bootstrap optimism-corrected changes in Harrell’s C-index. (C) Clinical-only and clinical-plus-score C-indices. (D) Likelihood-ratio tests for adding one continuous score term. These are exploratory association-level diagnostics, not prediction-model validation.
(TIF)

S1 Data Source data underlying the tables, figures, and numerical results, including the sample-level state/survival crosswalk and revision sensitivity-analysis inputs and outputs. (ZIP)

S2 File Analysis and figure-generation code, revision analysis code, and documentation of data sources, software requirements, and workflow provenance. (ZIP)
