# Supplementary material for: A public-data audit of transcriptomic prognostic transportability in R-CHOP-treated diffuse large B-cell lymphoma
Source: PLoS One. 2026 Oct 23;21(10):e0360937. doi: 10.1371/journal.pone.0360937 (PMC13693928; doi:10.1371/journal.pone.0360937)
Supplement: Supplementary file 1 [file pone.0360937.s001.docx]

S1 File. Supplementary methods and tables

**Supplementary methods**

## Outcome-blind state analysis

Discovery feature ranking and state assignment excluded survival and treatment labels. The discovery matrices comprised GSE10846 (420 DLBCL arrays), GSE93261 (149 follicular lymphoma arrays), and GSE21452 (64 mantle cell lymphoma arrays). Expression was mapped to Entrez genes, standardized by gene within each full cohort, and restricted to 18,067 genes measurable across the five state-analysis cohorts. The 2,000 genes with the best mean variance rank across the full discovery matrices were used. Sixty-four samples per discovery cohort were selected with seed 20260726, PCA was fitted to the pooled 192-sample balanced matrix, and k-means was evaluated for k=2-6 on principal components 1-6. The largest silhouette score selected k=4. All 420 GSE10846 arrays were subsequently assigned a state; among the 64 fitting arrays, 63 had known treatment and 34 entered the 232-patient R-CHOP OS analysis. All 414 known-treatment cases contributed to the descriptive state-by-treatment audit. The remaining GSE10846 arrays were not independent holdouts because they also contributed to feature ranking and standardization. GSE31312 (498 arrays) and GSE32018 (127 arrays) were used for external state projection/recovery. Stability was evaluated in 100 stratified 80% resamples; de novo external recovery independently refitted four clusters and matched expression centroids by exhaustive label permutations. Table R and state_discovery_survival_gsm_crosswalk.csv in S1 Data document the distinct sample roles.

## Probe and gene mapping

GPL570 probes were mapped to Entrez identifiers. Where multiple eligible probes represented one gene, the representative probe was selected by highest variance without using the relevant survival outcome. For GPL8432, exact Entrez mapping was used first and current-symbol fallback second. The highest-variance eligible probe across all 172 canonical GSE32918 patients was selected before the treated OS subset was modeled.

## Cox modeling

Cox models were fitted by Newton-Raphson optimization of the partial likelihood with Breslow handling of tied event times. Standard errors were obtained from the inverse observed information matrix. Hazard ratios and 95% confidence intervals were exponentiated from the score coefficient and coefficient plus or minus 1.96 standard errors. Complete cases were used for each model, which accounts for the lower adjusted N in GSE10846.

## Expanded meta-analysis

The original descriptive meta-analysis combined unadjusted continuous-score coefficients. Between-cohort variance was estimated by the DerSimonian-Laird method. Hartung-Knapp-Sidik-Jonkman inference used a t distribution with k minus 1 degrees of freedom, and the prediction interval used k minus 2 degrees of freedom. Cochran Q, its chi-square P value, tau-squared, I2, leave-one-cohort-out estimates, and each cohort's Q contribution and pooled-effect change were reported. A one-stage Cox model stratified by cohort tested score-by-cohort interaction. The original cohort-specific adjusted estimates were not pooled because their covariate sets differed. During revision, all four cohorts were also analyzed with an identical age (years divided by 10, linear term) and sex (male=1, female=0) adjustment. Eligibility and score scaling were unchanged, and all 929 cases had complete age and sex data. These adjusted coefficients were pooled using the same random-effects method. The 76-gene four-cohort summary includes both derivation cohorts and a 62-gene mapped-subset cohort, so it is a heterogeneity diagnostic rather than independent-validation evidence.

## Matched random-program null

The Affymetrix gene universe comprised genes measurable in GSE31312, GSE10846, and GSE87371 after outcome-blind highest-variance probe selection, excluding the 76 fixed genes. For each fixed position, a replacement was randomly selected from the 25 nearest eligible genes by standardized GSE87371 mean expression and log variance, constrained to the same GPL8432 mapping status and fixed direction sign. Genes were not reused within a program. All 1,000 programs therefore contained 76 genes, retained 62 genes in GSE32918, and preserved the 61 favorable/15 adverse sign pattern. Outcomes were not accessed during matching. Continuous Cox and meta-analysis estimates were then recomputed identically.

## Clinical incremental value

For the historical five-gene score, clinical-only models used age, sex, stage, ECOG, and COO in GSE31312; age, sex, stage, and ECOG in GSE10846; IPI and COO in GSE87371; and age, sex, and COO in GSE32918. The fixed-program analyses used IPI and COO in GSE87371 and age, sex, and COO in GSE32918. One score term was added to each base model. Likelihood-ratio tests compared nested models. Apparent Harrell C-indices and their increments were calculated, and 300 patient-level bootstrap samples estimated optimism in the increment. No cutoff, gene, sign, or weight was re-estimated.

## Coverage ablation

The full 76-gene score was calculated within GSE87371 and compared with a score retaining the 62 genes measurable in GSE32918, thereby omitting the 14 unmapped genes. One thousand uniform random 14-gene deletion sets were sampled without replacement. To construct the matched analysis, 12,000 random deletion sets were ranked by Euclidean distance between their omitted-gene mean/variance profile and that of the actual 14 omitted genes; the 1,000 closest sets were retained.

## Technical-replicate audit

GSE32918 titles without a replicate suffix defined the 172 canonical patient records. Noncanonical arrays were retained only for reliability analyses. Among 39 patients with replicates, the canonical score was compared with the mean replicate score using Pearson correlation, ICC(3,1), and Bland-Altman limits of agreement. For each of 1,000 resampling iterations, one available array was selected per patient, mapped-gene expression was re-standardized, and the unadjusted treated-cohort Cox model was refitted.

**Multiplicity and direction concordance**

The initial genome-wide discovery screen used a false-discovery-rate threshold of 0.05. Subsequent diagnostic analyses were not subject to a global multiplicity correction; their P values and matched-random percentiles are exploratory. Revision direction-concordance tests used two-sided binom.test(k, n, p=0.5) in R, with k/n=44/76 and 38/62, and Clopper-Pearson 95% intervals. Gene dependence is not modeled, so the binomial P values are descriptive independence-based benchmarks. Original one-sided binomial results in historical scripts are superseded for manuscript reporting by the explicitly two-sided revision results in Table U.

**Validation precision and power scenarios**

For a standardized continuous score and D=32 events, the approximation treats the test statistic as normal with unit variance and mean delta=log(HR)*sqrt(D). At two-sided alpha=0.05, power=Phi(-1.96-delta)+Phi(delta-1.96). Assumed effects were the derivation-cohort HRs, their inverse-variance pooled log HR, and hypothetical HRs of 1.30 and 1.50. The HR giving 80% power was solved numerically. This simplified unadjusted calculation assumes proportional hazards, non-informative censoring, and stable unit score variance in event risk sets; covariate correlation and model adjustment can reduce effective information. It is not a post hoc observed-power test based on the validation HR. Large derivation effects are selection-enriched and may substantially overstate plausible effects in new cohorts. The published confidence intervals remain the main description of validation precision. The method is based on Schoenfeld DA. Biometrics. 1983;39:499-503. doi:10.2307/2531021.

# Supplementary tables

Table A. Cohort provenance and analytic eligibility.

| **Cohort** | **Role** | **Platform** | **Raw arrays** | **Canonical** | **Eligible/known** | **Final OS N** | **Events** | **Endpoint unit** | **Duplicate handling** | **Key limitation** |
| --- | --- | --- | --- | --- | --- | --- | --- | --- | --- | --- |
| GSE31312 | 76-gene program derivation; not independent validation | Affymetrix GPL570 | 498 | 498 | 470 | 470 | 170 | years | No series-level replicate collapse required for final analytic records | Used for program selection; cannot serve as independent validation. |
| GSE10846 | 76-gene program derivation and treatment-context audit; not independent validation | Affymetrix GPL570 | 420 | 420 | 414 | 232 | 59 | years | No series-level replicate collapse required for final R-CHOP analytic records | CHOP and R-CHOP are historical treatment contexts, not randomized treatment arms. |
| GSE87371 | Independent full 76-gene validation | Affymetrix GPL570 | 223 | 223 | 109 | 87 | 32 | months | No series-level replicate collapse required | Restricted to R-CHOP14/21 and ABC/GCB records; no independent second full-coverage cohort in the current fixed workflow. |
| GSE32918 | 62/76 cross-platform sensitivity and technical-replicate audit; not full validation | Illumina GPL8432 HumanRef-8 WG-DASL | 249 | 172 | 140 | 140 | 62 | years | 249 arrays collapsed to 172 unsuffixed canonical patient titles; replicate reliability audited separately. | Only 62/76 genes map; outcome model is a coverage-limited sensitivity analysis. |

Table B. Cohort source, treatment composition, tissue context, and follow-up.

| **Cohort** | **Original publication** | **Tissue** | **Series treatment composition** | **Analysis population** | **Endpoint(s)** | **Clinical variables** | **Coverage** | **Median follow-up, y** |
| --- | --- | --- | --- | --- | --- | --- | --- | --- |
| GSE31312 | Visco et al., Leukemia 2012;26:2103-2113 | Formalin-fixed paraffin-embedded DLBCL tissue | R-CHOP consortium series; 498 arrays, 470 OS-linked records | R-CHOP-treated OS-eligible records | OS; PFS available | age; sex; stage; LDH; ECOG; IPI; extranodal sites; COO | 76/76 | 4.05 |
| GSE10846 | Lenz et al., N Engl J Med 2008;359:2313-2323 | Lymph-node biopsy; preservation not stated in GEO series metadata | 181 CHOP, 233 R-CHOP, 6 without complete treatment/outcome mapping | R-CHOP-like only | OS | age; sex; stage; LDH ratio; ECOG; extranodal sites; COO | 76/76 | 2.81 |
| GSE87371 | Dubois et al., Clin Cancer Res 2017;23:2232-2244 | DLBCL biopsy; preservation not stated in GEO series metadata | 76 R-CHOP21, 33 R-CHOP14, 13 R-Mini-CHOP21, 73 R-ACVBP+Conso, 11 R-ACVBP+ASCT, 17 ACVBP | R-CHOP14/21 only; ABC or GCB | OS; PFS available | age; sex; stage; IPI; COO; regimen | 76/76 | 4.31 |
| GSE32918 | Barrans et al., Br J Haematol 2012;159:441-453 | Formalin-fixed paraffin-embedded biopsy | 140 R-CHOP-treated patients and 32 not treated with curative intent; 249 arrays for 172 patients | R-CHOP-treated canonical patients | OS | age; sex; COO; treatment status | 62/76 | 5.85 |

Table C. Cross-cohort GSM overlap audit.

| **Comparison** | **Shared GSM IDs** | **Audit status** |
| --- | --- | --- |
| GSE31312 vs GSE10846 | 0 | No shared GSM identifiers |
| GSE31312 vs GSE87371 | 0 | No shared GSM identifiers |
| GSE31312 vs GSE32918 | 0 | No shared GSM identifiers |
| GSE10846 vs GSE87371 | 0 | No shared GSM identifiers |
| GSE10846 vs GSE32918 | 0 | No shared GSM identifiers |
| GSE87371 vs GSE32918 | 0 | No shared GSM identifiers |
| GSE32918 within-series | 0 | 249 arrays represent 172 canonical patients; 77 noncanonical technical-replicate arrays are explicitly retained only for reliability analysis. |

Table D. Historical direct five-gene continuous Cox models.

| **Cohort** | **Model** | **N** | **Events** | **HR/SD** | **95% CI** | **P** | **Gene directions** |
| --- | --- | --- | --- | --- | --- | --- | --- |
| GSE31312 | unadjusted | 470 | 170 | 1.394 | 1.207-1.609 | 5.868e-06 | 4/5 |
| GSE31312 | cohort_specific_adjusted | 470 | 170 | 1.220 | 1.033-1.442 | 0.01943 | 4/5 |
| GSE10846_RCHOP | unadjusted | 232 | 59 | 1.447 | 1.116-1.875 | 0.005276 | 3/5 |
| GSE10846_RCHOP | cohort_specific_adjusted | 206 | 52 | 1.251 | 0.948-1.651 | 0.1135 | 3/5 |
| GSE87371 | unadjusted | 87 | 32 | 1.628 | 1.139-2.327 | 0.007463 | 5/5 |
| GSE87371 | cohort_specific_adjusted | 87 | 32 | 1.414 | 0.780-2.565 | 0.2542 | 5/5 |
| GSE32918 | unadjusted | 140 | 62 | 1.054 | 0.816-1.361 | 0.6868 | 3/5 |
| GSE32918 | cohort_specific_adjusted | 140 | 62 | 0.957 | 0.722-1.267 | 0.7577 | 3/5 |

Table E. Fixed-program overall-survival Cox models.

| **Cohort** | **Coverage** | **Model** | **N** | **Events** | **HR/SD** | **95% CI** | **P** |
| --- | --- | --- | --- | --- | --- | --- | --- |
| GSE87371 | 76/76 | unadjusted | 87 | 32 | 1.232 | 0.868-1.750 | 0.2433 |
| GSE87371 | 76/76 | IPI_and_COO_adjusted | 87 | 32 | 0.883 | 0.556-1.404 | 0.5988 |
| GSE32918 | 62/76 | unadjusted | 140 | 62 | 1.130 | 0.894-1.429 | 0.3071 |
| GSE32918 | 62/76 | age_sex_COO_adjusted | 140 | 62 | 1.083 | 0.834-1.406 | 0.5495 |

Table F. Agreement between the GSE87371 full 76-gene and actual mapped 62-gene scores.

| **Spearman rho** | **ICC(3,1)** | **Quartile agreement** | **Top-quartile overlap** | **Genes** | **Omitted** | **Unadjusted HR** | **Change in log HR** |
| --- | --- | --- | --- | --- | --- | --- | --- |
| 0.990 | 0.992 | 0.908 | 0.833 | 62 | 14 | 1.191 | -0.0341 |

Table G. Percentiles of the actual mapped subset relative to random 62-of-76-gene subsets.

| **Random strategy** | **Metric** | **Actual 62** | **Random median** | **Random 2.5%-97.5%** | **Actual percentile** |
| --- | --- | --- | --- | --- | --- |
| mean variance matched random 62 of 76 | spearman_rho | 0.9903 | 0.9877 | 0.9807-0.9921 | 85.8 |
| mean variance matched random 62 of 76 | icc_3_1 | 0.9918 | 0.9905 | 0.9853-0.9935 | 74.7 |
| mean variance matched random 62 of 76 | quartile_agreement | 0.908 | 0.8851 | 0.8161-0.954 | 69.9 |
| mean variance matched random 62 of 76 | top_quartile_overlap | 0.8333 | 0.8333 | 0.76-1 | 35.6 |
| mean variance matched random 62 of 76 | cox_hr | 1.191 | 1.228 | 1.152-1.324 | 17.5 |
| mean variance matched random 62 of 76 | delta_log_hr_vs_full | -0.03407 | -0.003332 | -0.0676-0.07189 | 17.5 |
| uniform random 62 of 76 | spearman_rho | 0.9903 | 0.9875 | 0.9808-0.9921 | 85.4 |
| uniform random 62 of 76 | icc_3_1 | 0.9918 | 0.9904 | 0.9851-0.9938 | 74.1 |
| uniform random 62 of 76 | quartile_agreement | 0.908 | 0.8851 | 0.8161-0.954 | 70.7 |
| uniform random 62 of 76 | top_quartile_overlap | 0.8333 | 0.8333 | 0.76-1 | 38.5 |
| uniform random 62 of 76 | cox_hr | 1.191 | 1.227 | 1.145-1.317 | 20.0 |
| uniform random 62 of 76 | delta_log_hr_vs_full | -0.03407 | -0.004073 | -0.07316-0.06645 | 20.0 |

Table H. GSE32918 technical-replicate audit summary.

| **Metric** | **Value** |
| --- | --- |
| All arrays | 249 |
| Canonical patients | 172 |
| Noncanonical replicate arrays | 77 |
| Patients with replicates | 39 |
| Score ICC(3,1) | 0.611 |
| Score Pearson r | 0.664 |
| Bland-Altman mean difference | 0.163 |
| Bland-Altman limits | -0.514 to 0.840 |
| Median gene ICC(3,1) | 0.535 |
| Random-array HR median | 1.112 |
| Random-array HR 2.5%-97.5% | 1.062-1.169 |

Table I. Clinical positive controls.

| **Cohort** | **Control** | **N** | **Events** | **HR** | **95% CI** | **P** |
| --- | --- | --- | --- | --- | --- | --- |
| GSE31312 | IPI per point | 424 | 155 | 1.689 | 1.486-1.920 | 9.691e-16 |
| GSE87371 | IPI per point | 87 | 32 | 2.123 | 1.478-3.050 | 4.662e-05 |
| GSE10846_RCHOP | ABC versus GCB | 199 | 54 | 3.596 | 1.979-6.532 | 2.651e-05 |
| GSE87371 | ABC versus GCB | 87 | 32 | 2.234 | 1.056-4.726 | 0.03542 |
| GSE32918 | ABC versus GCB | 109 | 54 | 1.756 | 1.020-3.021 | 0.04203 |

Table J. Fixed 76-gene program and GSE32918 mapping status.

| **Entrez Gene ID** | **Symbol** | **Direction in score** | **Discovery beta** | **Mapped in GSE32918** |
| --- | --- | --- | --- | --- |
| 83451 | ABHD11 | Adverse | 0.2333 | Yes |
| 258 | AMBN | Adverse | 0.2603 | Yes |
| 222029 | DKFZp434L192 | Adverse | 0.2677 | No |
| 11072 | DUSP14 | Adverse | 0.3255 | Yes |
| 2917 | GRM7 | Adverse | 0.2299 | Yes |
| 105371079 | LOC105371079 | Adverse | 0.2654 | No |
| 55034 | MOCOS | Adverse | 0.3523 | Yes |
| 58498 | MYL7 | Adverse | 0.2346 | Yes |
| 105370333 | PCCA-DT | Adverse | 0.3033 | No |
| 284098 | PIGW | Adverse | 0.3057 | Yes |
| 170680 | PSORS1C2 | Adverse | 0.3613 | Yes |
| 126789 | PUSL1 | Adverse | 0.2769 | Yes |
| 159963 | SLC5A12 | Adverse | 0.2572 | Yes |
| 160728 | SLC5A8 | Adverse | 0.3151 | Yes |
| 23160 | WDR43 | Adverse | 0.2775 | No |
| 54806 | AHI1 | Favorable | -0.2177 | Yes |
| 79754 | ASB13 | Favorable | -0.2623 | Yes |
| 338758 | ATP2B1-AS1 | Favorable | -0.2225 | No |
| 965 | CD58 | Favorable | -0.3563 | Yes |
| 1000 | CDH2 | Favorable | -0.2408 | Yes |
| 8895 | CPNE3 | Favorable | -0.2758 | Yes |
| 220002 | CYB561A3 | Favorable | -0.2264 | Yes |
| 1540 | CYLD | Favorable | -0.2561 | Yes |
| 85479 | DNAJC5B | Favorable | -0.2389 | Yes |
| 23071 | ERP44 | Favorable | -0.2256 | Yes |
| 2260 | FGFR1 | Favorable | -0.2369 | Yes |
| 84248 | FYTTD1 | Favorable | -0.2420 | Yes |
| 9026 | HIP1R | Favorable | -0.2708 | Yes |
| 3120 | HLA-DQB2 | Favorable | -0.3515 | Yes |
| 9653 | HS2ST1 | Favorable | -0.3433 | Yes |
| 3702 | ITK | Favorable | -0.2789 | Yes |
| 3707 | ITPKB | Favorable | -0.3399 | Yes |
| 89857 | KLHL6 | Favorable | -0.2915 | No |
| 4005 | LMO2 | Favorable | -0.3955 | Yes |
| 105369746 | LOC105369746 | Favorable | -0.2860 | No |
| 105377468 | LOC105377468 | Favorable | -0.2235 | No |
| 4065 | LY75 | Favorable | -0.3624 | Yes |
| 4118 | MAL | Favorable | -0.2620 | Yes |
| 9448 | MAP4K4 | Favorable | -0.2935 | Yes |
| 23031 | MAST3 | Favorable | -0.2685 | No |
| 4753 | NELL2 | Favorable | -0.2654 | Yes |
| 10276 | NET1 | Favorable | -0.2767 | Yes |
| 8204 | NRIP1 | Favorable | -0.4470 | Yes |
| 284958 | NT5DC4 | Favorable | -0.2281 | No |
| 4947 | OAZ2 | Favorable | -0.3581 | No |
| 8481 | OFD1 | Favorable | -0.4143 | Yes |
| 91523 | PCED1B | Favorable | -0.4253 | Yes |
| 11201 | POLI | Favorable | -0.3454 | Yes |
| 5510 | PPP1R7 | Favorable | -0.3017 | Yes |
| 5636 | PRPSAP2 | Favorable | -0.2970 | Yes |
| 26191 | PTPN22 | Favorable | -0.2542 | Yes |
| 5788 | PTPRC | Favorable | -0.2948 | Yes |
| 54899 | PXK | Favorable | -0.3171 | Yes |
| 9475 | ROCK2 | Favorable | -0.3619 | Yes |
| 100507495 | SDCBP2-AS1 | Favorable | -0.2676 | No |
| 440829 | SHISA8 | Favorable | -0.2600 | No |
| 9187 | SLC24A1 | Favorable | -0.2864 | Yes |
| 162394 | SLFN5 | Favorable | -0.2501 | Yes |
| 10569 | SLU7 | Favorable | -0.2968 | Yes |
| 8773 | SNAP23 | Favorable | -0.2330 | Yes |
| 8082 | SSPN | Favorable | -0.3178 | Yes |
| 6819 | SULT1C2 | Favorable | -0.2803 | Yes |
| 55333 | SYNJ2BP | Favorable | -0.2742 | Yes |
| 7073 | TIAL1 | Favorable | -0.2536 | Yes |
| 7076 | TIMP1 | Favorable | -0.2810 | Yes |
| 338773 | TMEM119 | Favorable | -0.2363 | Yes |
| 81615 | TMEM163 | Favorable | -0.2942 | Yes |
| 7124 | TNF | Favorable | -0.3496 | Yes |
| 9760 | TOX | Favorable | -0.2860 | Yes |
| 100505501 | TOX-DT | Favorable | -0.2531 | No |
| 7185 | TRAF1 | Favorable | -0.3055 | Yes |
| 130507 | UBR3 | Favorable | -0.3486 | Yes |
| 55339 | WDR33 | Favorable | -0.2280 | Yes |
| 253461 | ZBTB38 | Favorable | -0.2699 | Yes |
| 55609 | ZNF280C | Favorable | -0.2385 | Yes |
| 147929 | ZNF565 | Favorable | -0.2341 | Yes |

Table K. Fixed 76-gene program relative to 1,000 outcome-blind matched random programs.

| **Metric** | **Fixed program** | **Random median** | **Random 2.5%-97.5%** | **Fixed percentile** |
| --- | --- | --- | --- | --- |
| GSE87371 HR | 1.232 | 0.936 | 0.688-1.253 | 96.8 |
| I2 (%) | 92.411 | 32.615 | 0.000-75.850 | 100.0 |
| Derivation-external beta gap | 0.678 | 0.272 | -0.026-0.581 | 99.5 |
| External full-cohort failure rate | 1.000 | 0.966 |  |  |

Table L. Quality of outcome-blind gene matching across random programs.

| **Programs** | **Genes/program** | **GPL8432 mapped** | **Mean distance, median** | **Mean distance, 95th percentile** | **Maximum distance, median** |
| --- | --- | --- | --- | --- | --- |
| 1000 | 76 | 62 | 0.076 | 0.083 | 0.430 |

Table M. Historical five-gene random-effects summary with Hartung-Knapp inference.

| **Cohorts** | **Pooled HR** | **HKSJ 95% CI** | **HKSJ P** | **Prediction interval** | **Q** | **Q P** | **I2 (%)** | **Tau2** |
| --- | --- | --- | --- | --- | --- | --- | --- | --- |
| 4 | 1.348 | 1.035-1.755 | 0.0367 | 0.767-2.370 | 5.115 | 0.1636 | 41.3 | 0.0103 |

Table N. Leave-one-cohort-out Hartung-Knapp analyses.

| **Omitted cohort** | **Pooled HR** | **HKSJ 95% CI** | **P** | **Prediction interval** | **I2 (%)** |
| --- | --- | --- | --- | --- | --- |
| GSE31312 | 1.330 | 0.762-2.321 | 0.1581 | 0.087-20.413 | 58.0 |
| GSE10846_RCHOP | 1.320 | 0.799-2.182 | 0.1405 | 0.124-14.104 | 58.4 |
| GSE87371 | 1.304 | 0.874-1.946 | 0.1041 | 0.209-8.118 | 49.9 |
| GSE32918 | 1.429 | 1.233-1.655 | 0.0090 | 0.926-2.205 | 0.0 |

Table O. Cohort influence on heterogeneity and the pooled effect.

| **Cohort** | **Q contribution** | **Pooled log-HR change if omitted** | **Absolute change** |
| --- | --- | --- | --- |
| GSE31312 | 0.155 | -0.0132 | 0.0132 |
| GSE10846_RCHOP | 0.251 | -0.0209 | 0.0209 |
| GSE87371 | 1.024 | -0.0331 | 0.0331 |
| GSE32918 | 3.685 | 0.0582 | 0.0582 |

Table P. One-stage cohort-stratified Cox model.

| **N** | **Events** | **Common HR** | **95% CI** | **Interaction chi-square** | **df** | **Interaction P** |
| --- | --- | --- | --- | --- | --- | --- |
| 929 | 323 | 1.355 | 1.217-1.508 | 5.140 | 3 | 0.1618 |

Table Q. Clinical-only versus clinical-plus-score incremental analyses.

| **Analysis** | **N** | **Events** | **Adjusted HR** | **95% CI** | **LRT P** | **Clinical C** | **Extended C** | **Delta C** | **Corrected delta C** |
| --- | --- | --- | --- | --- | --- | --- | --- | --- | --- |
| GSE31312 historical five-gene | 470 | 170 | 1.220 | 1.033-1.442 | 0.0192 | 0.668 | 0.679 | 0.011 | 0.008 |
| GSE10846 historical five-gene | 206 | 52 | 1.251 | 0.948-1.651 | 0.1125 | 0.701 | 0.720 | 0.020 | 0.013 |
| GSE87371 historical five-gene | 87 | 32 | 1.414 | 0.780-2.565 | 0.2472 | 0.743 | 0.760 | 0.017 | 0.006 |
| GSE32918 historical five-gene | 140 | 62 | 0.957 | 0.722-1.267 | 0.7587 | 0.677 | 0.675 | -0.002 | -0.009 |
| GSE87371 fixed 76-gene | 87 | 32 | 0.883 | 0.556-1.404 | 0.6004 | 0.743 | 0.749 | 0.006 | -0.004 |
| GSE32918 mapped 62/76 | 140 | 62 | 1.083 | 0.834-1.406 | 0.5542 | 0.677 | 0.678 | 0.001 | -0.007 |

# Supplementary software and reproducibility note

S2 File contains the author-generated analysis and figure-generation scripts and documentation. S1 Data contains the derived source tables and revision analysis inputs and outputs, including sample IDs and the fixed patient-level scores used for the new common-adjustment analyses. The supplied revision R script reproduces Tables S-V directly from these inputs. The original scripts document derivation-only gene selection and subsequent application, but neither code execution order nor local file modification times establish an independently verifiable pre-access lock. No externally timestamped preregistration or pre-access code deposit was available. Common-covariate, two-sided direction-concordance, and power analyses were added in response to peer review. Original numerical results were retained; a supplementary coverage-ablation description was corrected to state that the 14 unmapped genes were omitted and the 62 mapped genes retained.

**Table R. State-analysis roles and overlap with the survival cohorts.**

| **GEO cohort** | **State-assigned arrays** | **Balanced PCA/k-means fit** | **Known-treatment audit** | **R-CHOP OS cases** | **Fit / known treatment** | **Fit / OS** |
| --- | --- | --- | --- | --- | --- | --- |
| GSE10846 | 420 | 64 | 414 | 232 | 63 | 34 |
| GSE93261 | 149 | 64 | 0 | 0 | 0 | 0 |
| GSE21452 | 64 | 64 | 0 | 0 | 0 | 0 |
| GSE31312 | 498 | 0 | 0 | 470 | 0 | 0 |
| GSE32018 | 127 | 0 | 0 | 0 | 0 | 0 |
| GSE87371 | 0 | 0 | 0 | 87 | 0 | 0 |
| GSE32918 | 0 | 0 | 0 | 140 | 0 | 0 |

GSE10846, GSE93261, and GSE21452 supplied state-discovery expression data; the balanced fitting subset comprised 64 arrays per cohort. Full discovery matrices contributed to variance ranking and standardization. GSE31312 and GSE32018 were external state projection/recovery datasets; GSE31312 separately supplied survival-program derivation data. GSE87371 and GSE32918 were not used in the state analysis. The complete crosswalk in S1 Data contains 1,485 unique GSM records across state and OS roles. The two overlap columns count fitting arrays that also entered the indicated analysis.

**Table S. Common age- and sex-adjusted continuous-score Cox models.**

| **Score** | **Cohort** | **N (events)** | **HR/SD** | **95% CI** | **P** |
| --- | --- | --- | --- | --- | --- |
| Historical five-gene | GSE31312 | 470 (170) | 1.34 | 1.16-1.54 | 7.1e-05 |
| Historical five-gene | GSE10846_RCHOP | 232 (59) | 1.36 | 1.05-1.78 | 0.0218 |
| Historical five-gene | GSE87371 | 87 (32) | 1.31 | 0.90-1.89 | 0.158 |
| Historical five-gene | GSE32918 | 140 (62) | 1.04 | 0.81-1.33 | 0.758 |
| Fixed 76-gene / mapped subset | GSE31312 | 470 (170) | 2.20 | 1.87-2.59 | 1.56e-21 |
| Fixed 76-gene / mapped subset | GSE10846_RCHOP | 232 (59) | 2.74 | 2.10-3.58 | 1.8e-13 |
| Fixed 76-gene / mapped subset | GSE87371 | 87 (32) | 1.09 | 0.76-1.55 | 0.638 |
| Fixed 76-gene / mapped subset | GSE32918 | 140 (62) | 1.08 | 0.86-1.37 | 0.51 |

All models include linear age per decade and sex (male=1, female=0), with Breslow handling of ties and unchanged within-cohort score scaling. GSE31312 and GSE10846 are derivation cohorts for the 76-gene program; GSE32918 measures its 62-gene subset only. All 929 cases had complete age and sex data. P values are nominal and exploratory.

**Table T. Common age- and sex-adjusted random-effects summaries.**

| **Score** | **Pooled HR** | **HKSJ 95% CI** | **95% prediction interval** | **I2 (%)** | **P** |
| --- | --- | --- | --- | --- | --- |
| Historical five-gene | 1.27 | 1.05-1.54 | 0.94-1.72 | 8.8 | 0.027 |
| Fixed 76-gene / mapped subset | 1.65 | 0.77-3.53 | 0.19-14.10 | 92.7 | 0.128 |

Four cohorts were combined by DerSimonian-Laird between-cohort variance with Hartung-Knapp-Sidik-Jonkman inference (t, df=3); prediction intervals use t with df=2. The program summary includes two derivation cohorts and a mapped-subset cohort and is only a heterogeneity diagnostic, not independent-validation evidence.

**Table U. Two-sided gene-direction concordance tests.**

| **Cohort** | **Concordant / tested** | **Concordance (%)** | **Exact 95% CI (%)** | **Two-sided P** |
| --- | --- | --- | --- | --- |
| GSE87371 | 44/76 | 57.9 | 46.0-69.1 | 0.207 |
| GSE32918 | 38/62 | 61.3 | 48.1-73.4 | 0.098 |

Exact binomial tests use null probability 0.5 and two-sided alternatives; intervals are Clopper-Pearson intervals. P values are unadjusted and assume independent gene signs. Gene dependence limits formal inference; these are descriptive benchmarks. These two-sided results supersede one-sided calculations retained in the historical code archive.

**Table V. Effect-size scenarios for the 32-event full-coverage cohort.**

| **Assumed-effect source** | **Assumed HR/SD** | **Events** | **Two-sided alpha** | **Approximate power (%)** |
| --- | --- | --- | --- | --- |
| GSE31312 | 2.28 | 32 | 0.05 | 99.7 |
| GSE10846_RCHOP | 2.88 | 32 | 0.05 | >99.9 |
| Inverse-variance derivation summary | 2.43 | 32 | 0.05 | 99.9 |
| Smaller effect HR 1.3 | 1.30 | 32 | 0.05 | 31.7 |
| Smaller effect HR 1.5 | 1.50 | 32 | 0.05 | 63.1 |

The event-based continuous-score approximation assumes proportional hazards, non-informative censoring, and unit score variance. The 80%-power detectable HR is 1.64. The derivation summary combines log HRs by inverse-variance weighting. Derivation effects are selection-enriched, and adjustment/covariate correlation can reduce effective information. These are contextual scenarios, not observed-power tests based on the validation HR and not proof that smaller effects are absent.
